# Preferences and recommendations from content creators on carnivore diets: a social media analysis

**DOI:** 10.1186/s41043-026-01336-4

**Published:** 2026-05-17

**Authors:** Almiera Lietz, Janina Dapprich, Tobias Fischer

**Affiliations:** Center for Nutrition and Therapy (NuT), University of Applied Sciences Muenster, Corrensstraße 25, 48149 Muenster, Germany

**Keywords:** Carnivore, Carnivore diet, Lion diet, Social media, Content creator, Influencer, Meat

## Abstract

**Background:**

A carnivore diet is characterised by the exclusive consumption of animal foods, particularly red meat. Digital media, particularly TikTok and Instagram, often praise the health-promoting and disease-preventive properties of the carnivore diet. However, the scientific data on this form of nutrition is currently very limited.

**Methods:**

After creating a coding guide with an accompanying seven-day pretest and modification, a social media analysis was conducted on the Instagram platform over a period of one month. In addition to content related to nutrition and food, aspects such as lifestyle, advertising measures, and political or social statements were also collected. The survey was conducted quantitatively through categorization, accompanied by qualitative documentation of notable findings.

**Results:**

The analysis included 19 content creators (47% male, 53% female; aged 25–64) with an average of 157,758 ± 146,405 (25,200–582,000) followers. A total of 1,169 posts during the survey period showed a notable focus on health- and disease-related statements. With the exception of the strong emphasis on red meat, the nutritional and food recommendations were heterogeneous. This was accompanied by ideology-related themes, politically relevant statements, and critical portrayals of institutions such as science, politics, and industry, some of which could be classified as politically right-wing conservative. However, the data does not allow for a clear political classification. Overall, the carnivore diet was portrayed as positive.

**Conclusions:**

The one-sided view of carnivore nutrition, combined with political and social content, should be viewed critically. Nutrition professionals should pay attention to social media and counteract non-evidence-based claims with scientifically sound information.

**Supplementary Information:**

The online version contains supplementary material available at 10.1186/s41043-026-01336-4.

## Introduction

Every month, two billion people worldwide use Instagram to consume or post content [1]. Its technical features enable social feedback between content creators and consumers, which subsequently influences further actions of those involved [2]. It has been established that an increased level of identification with a character lead to a greater probability of emulating that character’s behaviours [3]. Parasocial relationships with content creators have been observed to enhance the perceived reliability of their statements [3, 4]. These findings could explain observed behaviour regarding nutrition-related information on social media platforms. For example, nutritional recommendations from content creators tend to be viewed as friendly, well-intentioned advice [5]. However, professionals address nutrition-specific topics less frequently than laypeople on social media [6–9]. Fernández et al. assessed misinformation on nutrition-specific topics in social media as a growing problem and identified audio-visual networks, such as Instagram, YouTube, and TikTok, as particularly associated with the spread of nutrition-related misinformation [10].

The #carnivorediet has been utilised in over 950,000 Instagram posts and #carnivore contains more than 2.6 million posts (July 23, 2025). A carnivore diet (CD) is characterised by the consumption of only animal products, the elimination of plant foods, and the avoidance of carbohydrates (CH) and highly processed foods. Currently, there is no uniform definition of a CD [11]. One of the most rigorous dietary regimens is the lion diet, which involves consuming only red meat, salt, and water [12]. Red meat also plays a fundamental role in other CD`s and is usually consumed with other food groups, such as white meat, eggs, fish, seafood, organ meats, animal fats, and fatty dairy products — in varying amounts and forms [11, 13, 14]. Advocates of this approach promise far-reaching health benefits, which they attribute to the elimination of supposedly harmful plant-based foods. The possible advantages of a CD include its elimination character, its ability to induce ketosis, and the enhanced bioavailability of carnivore foods [13]. However, these advantages contrast with prevailing recommendations that favour a balanced, plant-based diet for healthy individuals [15]. The CD lacks numerous plant compounds which are considered beneficial to health, such as bioactive substances and fibre [16–20]. Moreover, an elevated meat consumption is associated with the development of various diseases and reduced longevity [21].

Despite the growing popularity of CD`s on social media, knowledge in this young field of research is still limited. Digital media are a well-known source of nutrition-related information [5]. Vilhjalmur Stefansson, a Canadian researcher who undertook research expeditions in the Arctic and followed a CD for a year in the 1920s, like the Inuit did, is considered a prime example of the physiological safety of such a diet [22, 13]. In addition to Stefansson, there are numerous modern proponents of the CD [13]. Currently, there are hardly any significant clinical studies providing information on the short- and long-term effects of a CD. On social media disease- and health-specific claims backed by anecdotal evidence suggest that a CD can address a wide range of health issues [23, 12]. Therefore, it is not surprising that initial studies have shown that people primarily follow a CD for health and weight management reasons [14, 11].

It is presently unknown to what extent content about the CD influences followers’ eating habits and self-image. No quantitative data has yet been collected on the main topics of the carnivore movement on social media. However, such data could provide a basis for analysing the potential impact of engaging with carnivore content on eating habits. The aim of this social media analysis was to identify the main topics within the carnivore movement on Instagram in order to gain a better understanding. Additionally, dietary recommendations communicated by content creators were identified and reviewed.

## Materials and methods

### Sample selection

Prior to selecting the sample, a research account was created and trained using an algorithm with exclusively carnivore content. The following hashtags were used to focus the research account’s algorithm on carnivore topics: #plantfreemd, #carnivore, #thecarnivorelife, #meatbased, #liondiet, #meatheals, #carnivorewoman, #carnivorediet, #carnivorecommunity, #carnivorefitness, #howtodocarnivore, #carnivorerecipes, and #carnivoretribe. Furthermore, a search was conducted of the follower lists of suitable accounts to identify additional content creators. Taking into account the inclusion and exclusion criteria, up to 20 content creators were selected. The selection criteria were that content creators had to self-identify as carnivores and post regularly on the topic (minimum interval: twice a week). Gender, age, and other sociodemographic information were not selection criteria. The account had to be attributable to one single person, publicly accessible, and written in either German or English Additionally, the account needed to have at least 25,000 followers, which according to Kobilke’s definition corresponds to a ‘power middle-class influencer’ [24]. Content creators were excluded if they regularly consumed plant-based foods, except for coffee, alcohol, herbs, and spices.

### Ethics

The dataset for this study was obtained from publicly available Instagram accounts. After consultation with the Ethics Committee of the University of Applied Sciences Münster, no ethical approval was required. To ensure that the research was conducted in an ethical manner, all data was de-identified to protect the identities of the individuals and brands included.

### Development of the coding guide

A coding guide was created for quantitative data collection to ensure the validity, reliability, and objectivity of the data. The guide served as a tool for quantifying the objective characteristics of an entity. The goal was to enable each entity to be manually assigned to one or more categories as clearly as possible. Main categories and subcategories were created based on initial narrative research and observation of the sample. After creating a prototype coding guide, a one-week pretest was conducted to test and optimise it. During this seven-day period, all entities of the five most influential content creator in the sample were documented and transferred to the prototype coding guide. After modification, the following six main categories were created, each containing three to five subcategories and accompanying detailed categories: Body, Movement, and Exercise (a1–a4), Food (b1–b4), Nutrition (c1–c4), Politics and society (d1–d5), Lifestyle (e1–e3), and Advertising (f1–f4). For example, the main category “Lifestyle” included the subcategories “mental and psychological strength”, “sports and physical activity”, and “health-related recommendations”. Within the subcategory “sports and physical activity,” the detailed categories comprised “endurance training”, “strength training”, “general physical activity”, and “training in general” (see Supplementary, Table S1 and S2).

### Data collection and analysis

Data collection took place over a period of four weeks and included all Instagram posts and reels. Data was collected using text, image and video posts, and evaluated using the coding guide (see 2.2). Qualitative statements were documented in separate tables. During the coding process, the data was first assigned to the main categories and then to the subcategories, and finally to corresponding detailed categories. In addition to the post content, the total number of posts during the study period was documented. Furthermore, sociodemographic data, such as age, country of origin, and gender, was collected based on self-reported information or statements in the posts by the content creators. All evaluated posts were archived, and a second researcher independently reviewed the category assignment for plausibility, as part of a consensus-building process based on the coding guide. After recording the quantitative results, the qualitative anomalies were compiled. Each main category was examined for previously recorded quotes and comments. The total number of dietary recommendations was determined by combining the quantitative and qualitative data. Microsoft Excel 365 was used for data maintenance and evaluation, employing descriptive statistics such as mean, median, standard deviation, and percentages.

## Results

### General characteristics of content creators

A total of 19 content creators (47% male, 53% female; aged 25–64) were included in the analysis. One person was excluded due to an insufficient posting rate. Another account was included only up to day 14 of the survey, as the account was deleted for unknown reasons. The most frequently reported place of residence was the United States of America (USA; 57.9%), followed by the United Kingdom (UK; 15.8%) and Australia (AU; 10.5%). The place of residence of three content creators (15.8%) could not be identified. Apart from being content creators, their job titles included entrepreneur (26.3%), health coach (42.1%), medical doctor (10.5%), and nurse (5.3%). 36.8% were assigned to the “other” category, including retirees, homemakers, and people in professions unrelated to nutrition or health. Interestingly, 32% of content creators reported having had an eating disorder at least once in their lives. Meanwhile, 42% of the sample had tried a vegetarian diet. At the time of data collection, the median duration of the sample’s CD was 3.5 years (5.1 ± 5.4 years; 7.5 months − 24 years) (see Table 1). The median number of followers was 114,000 (157,758 ± 146,405; 25,200–582,000 followers).

A total of 1,169 posts were uploaded over the course of 29 days. On average, 32 (11–318) items were created per content creator, of which approximately 40% were reels and 60% were image formats. Posts were assigned to the following categories in descending order: Food (45.7%); Politics and Society (31.6%); Advertising (25.1%); Lifestyle (22.8%); Nutrition (17.1%); Other (14.8%); Body, Movement, and Exercise (12.5%; Table 2).

**Table 1:** Characteristics of the sample, including gender, previous nutrition, eating disorder, duration of CD, place of residence and occupational group

Characteristics	Value
Total sample Number of Followers (median; min, max) Male n (%) Female n (%) Age (min, max) Former eating disorder n (%) Former Vegetarian n (%) Duration of CD (median; min, max)	19 114,000 (25,200–582,000) 9 (47%) 10 (53%) 25–64 years 6 (32%) 8 (42%) 3.5 years (0.6–24 years)
**Place of residence**	
USA UK AU unidentified	57.9% 15.8% 10.5% 15.8%
Occupational group*	
Medical doctor Nurse Entrepreneur Health coach (Exercise or nutrition) other	10.5% 5.3% 26.3% 42.1% 36.8%

* Multiple assignments possible

### Body, movement, and exercise

Of the 1,169 items, 146 (12.5%) were assigned to the main category ‘body, movement, and exercise’, which had 176 content hits in its subcategories. The most frequently represented content was related to athletic activity (*n* = 76; 43.2%) and selfies showing bare skin (*n* = 66; 37.5%). The subcategories ‘selfie’ und ‘beauty’ each accounted for 9.7% (*n* = 17). Strength training (59.3%) and endurance sports (23.3%) were of particular interest within the detailed categories. In this context, the exposed abdomen (24.2%), chest (18.8%), and legs were frequently depicted (14.5%). Other body parts were less relevant.

### Food

Most content assignments (*n* = 534; 45.7%) were in the main category ‘food’, with a total of 809 content assignments in the subcategories. The depiction of individual foods (*n* = 532; 65.8%) was highly prevalent. The focus here was primarily on red meat (33.2%), eggs (13.5%), dairy products (11.1%), and animal fats (9.6%). Organ meats (1.3%), animal broth (1.2%), bones (0.8%), and dietary supplements (0.7%) were rarely shown. The most frequently preparation method was pan-frying (23.4%), followed by grilling (14.2%), baking (13.8%), air frying (6.9%), and boiling (6.0%). The majority of the meals featured (48.4%) were not assigned to any conventional daily meal by the content creators. Overall, food and drink were mainly presented in video form.

### Nutrition

A total of 200 items (17.1%) with 329 content hits across the subcategories were assigned to the main category ‘nutrition’. Of particular relevance were references to health (*n* = 134; 40.7%) and disease (*n* = 127; 38.6%) in connection with a CD. Aspects of fitness (*n* = 50; 15.2%) and nutritional supplements (*n* = 18; 5.5%) were mentioned less frequently. Regarding health claims, all statements were positively associated with a CD. The most common statements were those regarding an improved general condition (26.7%) as a result of a meat-based diet. Statements regarding increased energy and mental health (13.2%), improved digestion (6.2%), increased satiety (5.9%), and a more stable hormone balance (5.9%) were also prevalent. In relation to diseases, weight loss (36.8%) was the most frequently mentioned, followed by skepticism toward scientific critism (10.3%), inflammation (6.3%), autoimmune diseases (5.8%), and employing a CD to treat type I and II diabetes mellitus (5.4%). Within the subcategory of fitness, the importance of protein quality and quantity (34.1%), muscle growth (30.5%), regeneration (14.6%), and increased testosterone (7.3%) through a CD were highlighted. Most statements about dietary supplements expressed the opinion that supplementation is unnecessary during a CD (25%). This contrasts with recommendations for electrolytes (16.7%), magnesium (12.5%), protein powder (8.3%), and individualised supplementation (8.3%).

### Politics and society

In the main category ‘politics and society’, 369 (31.6%) posts with 534 hits were sorted into subcategories. Warnings were issued most frequently (49.1%), followed by memes with political and social content (23.6%) or opinions on other diets (16.7%). Environmental issues (6.6%) and economic issues (4.1%) were found to be less relevant. The warnings rejected the consumption of (highly) processed foods (26.8%), followed by carbohydrates (14.8%), plant-based foods (11.0%), and various chemicals and toxins (10.7%). Furthermore, negative portrayals of politics and the pharmaceutical industry (9.4%), science (7.1%) and the food industry (5.4%) were identified. Other diets besides the CD were portrayed neutrally, with the exception of vegetarianism. More than half of the hits (54.5%) in this subcategory made negative references to vegetarianism. The other diets mentioned were animal-based diets (19.2%), ketogenic diets (14.1%), low-carb diets (6.1%), and ketovore diets (3%). Regarding the environment, statements were made about animal production and agricultural systems (48.8%), climate change downplaying (30.2%), animal ethics (16.3%), and, in one case, raising awareness of food waste (4.7%). Economically, the CD was predominantly classified as inexpensive (59.1%).

### Lifestyle

Of the 267 (22.8%) posts in the main category ‘lifestyle’, 427 hits were identified in the related subcategories. The subcategories of mental and psychological strength (37.2%), health-related recommendations (36.3%), and exercise and movement (26.5%) were almost equally relevant. Motivation and motivational content for achieving goals (36.9%) played a particularly important role in the area of mental and psychological strength. Stress and coping with stress (12.9%), optimism (12.0%), and coping with negative overload (9.2%) were also significant topics. Lifestyle-related physical activity was most frequently discussed in connection with general movement (30.0%), strength training (26.4%), and a reference to training in general (25.0%). The health-related recommendations subcategory included all lifestyle recommendations intended to promote health that did not explicitly refer to a CD or exercise. These included non-carnivore dietary recommendations (23.8%), maintaining interpersonal relationships (16.0%), body awareness (9.4%), having a balanced sleeping pattern (9.4%), and sunbathing (7.0%). Less frequently mentioned were macronutrient tracking (6.6%), meal prepping (4.9%), not weighning oneself too frequently (4.5%), avoiding alcohol consumption (4.1%), cold showers (3.7%), infrared light (3.3%), meditation (2.9%), fasting (2.9%), and frequency healing (1.6%).

### Advertising

A total of 294 (25.1%) posts were assigned to the main category ‘advertising’, with 356 hits in the subcategories. Media formats were advertised most frequently (*n* = 185; 52.0%), followed by advertising for food (-producers) (*n* = 64; 18%) and for coaching and counselling programs (*n* = 45; 11.8%). Advertising for dietary supplements (*n* = 19; 5.4%) and body care products (*n* = 8; 1.8%) were less common. 51.8% of the hits for media advertising were related to various websites, followed by advertising for books (32.7%), podcasts (10.5%), YouTube (3.6%), and Patreon (0.9%). Among food producers, meat processing companies were the most commonly advertised (31.5%), followed by processed foods (23.4%), salt and processed ingredients (11.7%), and unprocessed foods (9.9%). The most commonly advertised dietary supplements were protein powders (16.7%), electrolytes (8.3%), vitamins and minerals (4.2%), collagen (4.2%), and enzymes (4.2%) (see Table 2).

**Table 2:** The number of assigned posts and hits in the main (n), sub (n; %) and detailed (n; %) categories. The detailed categories refer to the number and percentage of hits within the subcategory. Multiple categorizations were possible in all categories except the subcategory “Eating and drinking”

Categories	Number of posts Main category (*n*)	Number of hits Subcategory (*n*; %)	Number of hits detailed categories (*n*; %)
**Body, movement and exercise**	146	176	314
Selfie		17 (9.7%)	18 (5.7%)
Selfie with exposed skin		66 (37.5%)	186 (59.2%)
Presentation of the body during sports		76 (43.2%)	86 (27.4%)
Beauty		17 (9.7%)	24 (7.6%)
**Food**	534	809	1560
Food diary		120 (14.8%)	128 (8.2%)
Eating and drinking		84 (10.4%)	84 (5.4%)
Preparation		73 (9.0%)	218 (14.0%)
Food		532 (65.8%)	1130 (72.4%)
**Nutrition**	200	329	602
Illness-related		127 (38.6%)	223 (37.0%)
Health-related		134 (40.7%)	273 (45.3%)
Fitness-related		50 (15.2%)	82 (13.6%)
Dietary supplements		18 (5.5%)	24 (4.0%)
**Politics and society**	369	534	700
Environment		35 (6.6%)	43 (6.1%)
Economy		22 (4.1%)	22 (3.1%)
Ether diets		89 (16.7%)	99 (14.1%)
Memes		126 (23.6%)	144 (20.6%)
Warning		262 (49.1%)	392 (56.0%)
**Life style**	267	427	633
Mental und psychological strength		159 (37.2%)	249 (39.3%)
Exercise and movement		113 (26.5%)	140 (22.1%)
Health-related recommendations		155 (36.3%)	244 (38.5%)
**Advertising**	294	356	488
Media formats		185 (52.0%)	220 (45.1%)
Dietary supplements		19 (5.3%)	24 (4.9%)
Food (-producers)		64 (18.0%)	111 (22.7%)
Body care products		8 (2.2%)	8 (1.6%)
Coaching and counselling programs		42 (11.8%)	42 (8.6%)
Other		38 (10.7%)	83 (17.0%)
Other	173	173	173
Total	1983	2804	4470

### Extraction of recommendations on carnivore nutrition

Based on quantitative data collection and qualitative content analysis, the following dietary recommendations were extracted from the sample in descending order of frequency:

1. High-fat red meat should form the basis of a CD.
2. Eggs and animal fats are important sources of nutrients after red meat.
3. White and processed meats can be consumed. When it comes to processed meat, choose less processed varieties.
4. Dairy products can be included in the diet. Dairy products with a high fat content and low carbohydrate content are preferred. Raw dairy products are recommended.
5. Fish, seafood, shellfish, and organ meats can also be added to the diet.
6. Do not consume highly processed foods.
7. Do not consume plant-based and carbohydrate-rich foods.
8. Do not consume seed oils.
9. When choosing foods, prefer the following: pasture-raised animal products, organically produced foods, wild-caught fish, and products from grass-fed animals.
10. Consume enough salt.
11. Dietary supplements are usually unnecessary.
12. A CD can be used as an elimination diet.

## Discussion

Overall, the nutritional recommendations in the sample were heterogeneous and showed contradictions between the content creators. Accordingly, the recommendations depend heavily on the content creator. One consistent recommendation was the use of red meat, or a positive emphasis on it. Therefore, the red meat was the most frequently discussed food. This is consistent with a survey of a total of 2,029 carnivores, which also identified red meat as the most frequently consumed food [11]. Other animal-based foods, such as eggs and milk, played a less prominent role, which also aligns with the findings of Klement and Matzat and Lennerz et al. regarding inconsistent consumption in the context of a CD [14, 11].

Recommendations for incorporating other food groups vary. In particular, fish, seafood, shellfish, and organ meats were of little relevance in the present sample and were rarely discussed. This contradicts with the results of Lennerz et al. and Klement and Matzat [11, 14]. In the survey by Lennerz et al., a total of 42% of participants reported consuming organ meats weekly or more frequently [11], and in Klement and Matzat’s explorative study, 92% of participants consumed organ meats regularly [14]. One possible explanation for this discrepancy could be the social desirability on social media. Organ meats in particular do not play a significant role in the diet of many Western diets, and their consumption is sometimes viewed negatively [25–27]. Fish and seafood were also less relevant in Lennerz et al. [11]. However, Klement and Matzat found consumption of fish and seafood in 54% of cases, which again points out the heterogeneous dietary practices [14].

The level of knowledge about the potential benefits and risks of a CD, as well as the understanding of the carnivore lifestyle, is heterogeneous and incomplete. Additionally, it has been shown that lifestyle and relating factors, such as health and well-being, also play an important role. From a nutritional perspective, food choices in a CD tend to correspond to a high-risk dietary pattern due to the high consumption of animal-based foods and the avoidance of plant-based foods. Consuming meat products is often linked with an elevated risk of diet-related illnesses and a higher number of disability-adjusted life years [28, 29]. Paradoxically, the main motivation for many carnivores is maintaining and achieving good health [11, 14]. In addition, there are growing reports of individuals who feel better as a result of a CD [11, 30, 14]. Other reasons for a CD include curiosity (17%), ethics (4%), and others (4%) [14].

According to the collected data and the derived dietary recommendations, the CD is a high-fat, high-protein animal-based diet which excludes the consumption of plant-based and (highly) processed foods. The resulting pattern of macronutrients positions the CD as a low-carb-high-fat (LCHF) diet, which, depending on how it is practiced, can potentially induce ketosis. In most cases, it can be assumed that a low level of ketosis will be achieved, which differs from or is likely to fall below the levels achieved with the well-known clinically applied ketogenic diets [31]. There are no known recommendations regarding the proportion of energy that should be consumed from fat, protein or carbohydrates in a CD. However, Lennerz found that 41% of participants aimed for ketosis or interpreted the diet as ketogenic, whereas Klement and Matzat found that this applied to 62.5% of participants [11, 14]. Due to the high protein intake, it can be assumed that the resulting increase in gluconeogenesis either counteracts ketogenesis or results in only low concentrations of ketone bodies in the blood. Therefore, a possible explanation for the reported positive effects of a CD, such as weight loss, improvement of autoimmune and diabetic diseases, and anti-inflammatory effects could be based on an LCHF or ketogenic approach [32–35]. The CD’s high protein content could also be responsible for the weight loss, as people tend to eat less at subsequent meals after consuming a protein-rich meal, leading to lower energy intake [36, 37].

In this context the survey by Lennerz et al. as well as a case series, reported weight loss as a result of a CD [11, 30]. Additionally, food cravings decreased, and a high level of satiety was reported [11]. The carnivore lifestyle, which emphasises health-related topics such as sports, exercise, and mental health, coupled with limited food selection, could - additional to ketogenic-related metabolic mechanisms - contribute to a negative calorie balance, which is a key factor in weight loss. Thus, the health improvements could be partially due to the placebo effect. A firm belief in the health benefits of a diet or lifestyle can encourage behavioural changes that actually have a positive effect on health. For instance, individuals convinced that a vegetarian diet aids in weight loss are more likely to adhere to their diet, potentially promoting real weight loss [38]. The present social media analysis is consistent with these statements, as weight loss was a popular topic among the sample as well as frequent discussions about hunger and satiety. In addition to health-related motives, the simplicity of meal preparation may further encourage adoption of the CD, as its limited food range reduces the need for complex planning [39]. Taken together, these factors highlight the tension between perceived benefits and the potential health risks suggested by current evidence.

Another potential benefit of a CD is an alteration in certain blood parameters. Lennerz et al. documented self-reported improvements in c-reactive protein (CRP), gamma-glutamyltransferase (GGT), and triglyceride/HDL ratio. According to the participants’ reports, they experienced improved insulin resistance and reduced their diabetes medication. However, triglyceride and LDL-C levels increased [11]. In the case series by Norwitz et al., elevated LDL-C and total cholesterol levels were observed as well [30], which is consistent with Klement and Matzat’s findings [14]. These results may be due to the LCHF or ketogenic nature of the CD. For example, a meta-analysis linked a very low-calorie ketogenic diet with a reduction in triglycerides and GGT [40]. Wang et al. concluded in a meta-analysis that a ketogenic diet can reduce triglycerides, blood pressure, and body weight and increase HDL-C. They also found reduced blood glucose and insulin concentrations compared to control groups. However, no significant improvement in HbA1c levels was observed, and an increase in total cholesterol and LDL-C was noted [41]. Further clinical studies have also shown significant increases in LDL-C and ApoB levels in the context of a ketogenic diet [42–44]. Furthermore, some variants of the CD emphasise unprocessed meat and animal quality, both of which can influence its nutritional profile. For example, grass-fed meat generally contains more omega-3 fatty acids and a more favourable omega-6/omega-3 ratio than conventionally produced meat [45, 46]. The traditional Inuit diet is often cited in this context, as it consisted largely of raw animal foods and was associated with generally good health outcomes, partly due to its fatty acid profile and micronutrient content [47–49]. However, these findings are only of limited relevance to modern populations, particularly given differences in lifestyle, food availability, and physical activity. Many chronic diseases develop over decades, so current evidence is insufficient to assess the long-term health effects of the CD. Available studies lack clinical endpoints, and prospective longitudinal data are missing. Given its nutrient profile, potential deficiencies may increase long-term health risks. This need for caution is supported by a long-term follow-up study reporting a potential U-shaped association between carbohydrate intake and mortality [50]. Overall, it should be noted that there is an assumed link between meat consumption and an increased risk of developing diabetes, cancer, obesity, hypertension, inflammation and atherosclerosis. However, it is difficult to transfer this to CD`s due to a lack of data [21].

Phelan et al. demonstrated that the CD had the lowest score in a comparison of seven trending diets using Healthy Eating Index (HEI) scores. This was due to the elimination of plant-based foods and the high content of saturated fatty acids and sodium. In addition to fibre, micronutrients such as potassium, calcium, and vitamin D were also classified as potentially deficient [51]. Goedeke et al. determined that thiamine, magnesium, calcium, vitamin C, iron, folate, iodine, and fibre are potentially critical in the carnivore diet. Including liver and iodised salt in hypothetical diet plans for women was necessary to ensure adequate iron and iodine intake [52]. In contrast, O’Hearn’s hypothesis proposes different micronutrient requirements for a CD due to reduced carbohydrate metabolism, microbiome modulation, and increased meat intake [13]. In summary, despite the high bioavailability of animal-based foods, a CD appears to be associated with micronutrient deficiencies, contradicting content creators’ claims that such a diet predominantly meets nutritional requirements. Additionally, the advantages of the CD as an elimination diet do not outweigh the risk of nutritional deficiencies. Less risky elimination diets (e.g. the low-FODMAP diet) should therefore be preferred.

Muller et al. conducted a social media analysis examining the posting behaviour of two carnivore content creators from 2020 to 2023 in terms of their rhetoric. The analysis revealed a proliferation of conspiracy theories and right-wing ideologies in the content creators’ content. They viewed and ideologised meat consumption as a means of achieving “ultimate white masculinity.” Although the carnivore community is heterogeneous, Muller et al. note the potential for less ideologically driven content creators to provide a shallow entry point into the community, and through their networks, these content creators could facilitate a path into more radical, racist-oriented scenes [53]. The social rejection identified by Protogerou et al. outside the community could further reinforce this effect [54]. Monteiro et al., described carnism as an ideology and found a connection between meat consumption and carnistic beliefs. These beliefs justify the killing of animals for food and counteract cognitive dissonance [55]. Cognitive dissonance can be triggered by the “meat paradox,” which expresses a dilemma between meat consumption and empathy for animals [56]. Monteiro et al. examined, carnistic defence, which legitimises the consumption of animals and trivialises their suffering. They also analysed carnistic dominance, which justifies killing animals for food based on hierarchical superiority, also known as speciesism. Both concepts are linked to socio-political attitudes, such as right-wing authoritarianism and social dominance orientation. Carnist dominance has also been associated with symbolic racism and sexism [55]. The present analysis revealed tendencies toward conspiracy-related narratives, ideological elements, and politically relevant statements that may support the justification of the promoted dietary practices. While right-wing extremist views identified in Muller et al.’s social media analysis were generally absent from the present sample, some politically relevant and ideology-related themes were identified [53]. Furthermore, the present analysis showed that four of the eight carnistic beliefs described by Monteiro et al. could be identified, indicating that the content creators implement these beliefs to justify the consumption of animal-based foods [55]. However, the present findings are insufficient to classify the sample within a specific political spectrum.

Food carries not only nutritional relevance but also substantial cultural, social, and moral significance [57, 58]. In this context, dietary practices cannot be understood solely as individual choices but must be considered within their broader social environments. Protogerou et al. demonstrated that individuals adhering to a carbohydrate-free diet frequently experience social conflicts, both in personal relationships and in interactions with healthcare professionals outside their respective communities. In some cases, social pressure even led participants to consume plant-based foods despite their dietary intentions [54]. At the same time, social media has become increasingly influential in shaping dietary orientations and practices. Leismann and Godemann described social media as a way for followers to connect with each other in order to identify their ideal diet [5]. Social interactions via various online platforms can significantly influence dietary behaviour [54]. Content shared ranges from evidence-based nutritional information and recipes to tailored dietary advice for specific target groups [59]. Notably, discussions around “healthy eating” are often characterized by normative frameworks and strict dietary rules, whereas topics such as weight loss tend to be shaped by social support within online communities [60]. Furthermore, popular dietary trends such as vegan or ketogenic diets dominate online discourse, with content creators and users influence public perceptions through engaging and often emotionally driven narratives [61, 62]. Different platform formats serve distinct functions: short-form platforms such as TikTok and Instagram facilitate the rapid dissemination of trends, while long-form platforms like YouTube are more suitable for in-depth educational content [63]. Overall, these findings suggest that the uncritical idealization of dietary patterns on social media may contribute to the perception of certain diets as optimal. In the context of the present study, this dynamic may partly explain the appeal of such dietary practices. Regardless of the nutritional approach, professionals should be aware of social media’s potential influence on recipients’ nutritional decisions and self-identification [64].

A potential limitation of this social media analysis is a selection bias due to the sample size and selection process. In the present sample, female content creators were slightly overrepresented compared to male content creators, despite the fact that high levels of meat consumption are more commonly associated with men in the literature [65–67]. This pattern may be partly explained by the use of hashtags such as #carnivorewoman, which can influence algorithmic visibility and content selection. Additionally, women tend to be highly active as influencers across various domains [68–70] and often achieve higher levels of engagement with their content [71]. Furthermore the content creators are exclusively from English-speaking countries and the platform Instagram. Additionally, the sample only represents a portion of the total number of content creators in the carnivore community, which may affect its representativeness. In this context, it should be noted that the total number of carnivore content creators is unknown and cannot be determined. A further limitation relates to the algorithm-based sampling strategy of the research account, as platform algorithms tend to prioritize highly engaging, similar, and potentially more polarizing or popular content, which may have led to an overrepresentation of certain themes in the sample. Due to the amount of information shared on social media, it was impossible to analyse every topic in depth. This limitation was addressed by focusing on specific topics in advance. To avoid confirmation bias, the category guide was formulated as precisely as possible and included many main, sub, and detailed categories. Moreover, the items were checked for plausibility after categorization. As the source material was image- and video-based, subjective influences in the categorisation process cannot be completely ruled out.

## Conclusion

Overall, within the analysed sample, posts frequently focused on the potential health benefits of CD`s for treating illnesses, and such benefits were reported exclusively in a positive manner by the content creators. Red meat appeared to be the most important food, whereas nutritional practices and recommendations were heterogeneous. This was accompanied by ideology-related themes, politically relevant statements, and narratives expressing distrust toward institutions such as science, politics, and industry, some of which can be attributed to the politically conservative right-wing spectrum. However, these observations should be interpreted with caution, as the exploratory design of this study does not allow for broader generalizations or clear political classification. Regarding the CD, there are numerous research gaps concerning its potential benefits, risks, and long-term use. These gaps severely limit the scientific evaluation of this form of nutrition. Given the prominent role of social media in nutrition communication, future research should continue to examine how dietary patterns such as the CD are presented online. Nutrition professionals may also benefit from paying closer attention to such content and providing scientifically evidence-based nutritional information to the general public.

## Supplementary Information

Below is the link to the electronic supplementary material.

Supplementary Material 1.

## Data Availability

The raw data (German language) supporting the conclusions of this article will be made available by the authors on request.
