# Supplementary material for: Preferences and recommendations from content creators on carnivore diets: a social media analysis
Source: J Health Popul Nutr. 2026 May 17;45:137. doi: 10.1186/s41043-026-01336-4 (PMC13195945; doi:10.1186/s41043-026-01336-4)
Supplement: Supplementary file 1 — Supplementary Material 1. [file 41043_2026_1336_MOESM1_ESM.docx]

***Supplementary***

***Table S1*** *Overview of the main categories with corresponding subcategories, coding numbers, definitions, and examples of categorization.*

| **Subcategories** | **Coding** | **Definition** | **Examples** |  |
| --- | --- | --- | --- | --- |
|  |  |  |  |  |
| **Body, movement and exercise** | | | |  |
| Selfie | a1 | Depiction of oneself for the purpose of showcasing one's body. This category includes depictions of the body that aim to present it in a positive light. This includes poses that emphasize the body or a post description that refers to the appearance of the body. | A photo of the influencer showing off their slim figure in a posed body position. |  |
|  |  |  |  |  |
|  |  |  |  |  |
|  |  |  |  |  |
| Selfie with exposed skin | a2 | Presentation of oneself with bare skin to showcase the corresponding body part. The post is sorted into this category if the bare body part is explicitly the focus of the presentation. | The influencer wears a T-shirt and flexes their arm muscles to show them off. |  |
|  |  |  |  |  |
|  |  |  |  |  |
| Presentation of the body during sports | a3 | All depictions of the body during sports. Posts that suggest that the influencer is doing sports are sorted into this category. A distinction is made between endurance, strength training, and exercise that goes beyond everyday activity. | A person films themselves doing strength training at the gym. |  |
|  |  |  |  |  |
|  |  |  |  |  |
|  |  |  |  |  |
| Beauty | a4 | Posts that associate beauty with a carnivorous diet. This category distinguishes between the claimed benefits of a carnivorous diet for typical ideals of beauty. | The influencer says that the carnivorous diet stimulates hair growth. |  |
|  |  |  |  |  |
|  |  |  |  |  |
| **Food** | | | |  |
| Food diary | b1 | Presentation of all foods consumed by the influencer during a specific meal. A distinction is made between breakfast, lunch, snacks, and dinner. The post falls into this category if the meal is named. | The influencer shows all the foods they consumed for breakfast. |  |
|  |  |  |  |  |
|  |  |  |  |  |
|  |  |  |  |  |
| Eating and drinking | b2 | Depiction of a person eating or drinking. A post is classified in this category if the act of eating or drinking is recognizable. | A photo showing the influencer biting into a piece of meat. |  |
|  |  |  |  |  |
| Preparation | b3 | Depiction of a carnivorous recipe or demonstration of food preparation. The post is classified in this category if it either clearly shows the method of preparation of a recipe or presents a method of preparation. | The influencer shows how they use their pan to prepare ground beef. |  |
|  |  |  |  |  |
|  |  |  |  |  |
| Food | b4 | As soon as a food is presented or is the thematic focus of the post, it is sorted into this category. Only carnivorous foods and dietary supplements are included in this category. Foods that are placed in a context that does not recommend their consumption are not sorted into this category. | The influencer shows what beef kidneys look like and what nutrients they contain. |  |
|  |  |  |  |  |
|  |  |  |  |  |
|  |  |  |  |  |
|  |  |  |  |  |
| **Nutrition** | | | |  |
| Illness-related | c1 | All posts that place a carnivorous diet in a health-promoting or therapeutic context in relation to diseases are sorted here and differentiated according to clinical pictures. Exceptions are the subcategories of *switching to a carnivorous diet* and *doubts about scientific criticism.* | The influencer describes their symptomatic improvement in migraine through the carnivorous diet. |  |
|  |  |  |  |  |
| Health-related | c2 | All posts that make health-related statements in the context of the carnivorous diet. This category distinguishes between the health-related benefits of the carnivorous diet. | A person describes how the carnivorous diet increases mental performance. |  |
|  |  |  |  |  |
|  |  |  |  |  |
| Fitness-related | c3 | All posts that make fitness-related statements in a nutritional context. The fitness reference here refers to sporting activities. The post is also sorted here if the connection to sport and fitness is not explicitly described, but is generally associated with the central statement. E.g., the protein quality of a meal. | A person shows themselves jogging and says that they can run further thanks to the carnivore diet. |  |
|  |  |  |  |  |
|  |  |  |  |  |
|  |  |  |  |  |
| Dietary supplements | c4 | All posts that discuss nutritional supplements in the context of the carnivore diet. If a person takes a supplement without discussing it in the context of the carnivore diet, the corresponding entity is also sorted into this category. | A person explains that supplementing with dietary supplements is not necessary in the carnivore diet. |  |
|  |  |  |  |  |
|  |  |  |  |  |
|  |  |  |  |  |
|  |  |  |  |  |
| **Politics and society** | | | |  |
| Environment | d1 | Entities that discuss various environmental aspects related to the carnivorous diet. This category covers the sustainability aspects of the environment, animal welfare, and social responsibility. | A graphic comparing the water consumption of grazing animals with the water consumption of avocados. |  |
|  |  |  |  |  |
|  |  |  |  |  |
| Economy | d2 | Posts that address the economic aspects of a carnivorous diet. | The influencer shows how expensive their weekly shopping was. |  |
| Other diets | d3 | Posts that discuss different diets. A post is sorted into this category if a diet other than the carnivorous diet is mentioned or if the content of the post suggests that it refers to a different diet. | A meme that addresses the risk of nutritional deficiencies in vegetarianism. |  |
|  |  |  |  |  |
|  |  |  |  |  |
|  |  |  |  |  |
| Memes | d4 | Posts that meme a carnivorous diet in a positive or neutral context. Alternatively, posts that meme non-carnivorous content in a negative or satirical context. A meme is defined as a satirical treatment of a topic in pictorial or videographic form. | A satirical graphic that portrays meat consumption in a positive light. |  |
|  |  |  |  |  |
| Warning | d5 | Warning against the consumption of a food, medicine, industry, or activity. If the post explicitly warns against the named aspects or places them in a negative context, it is classified in this category. | Warning against the consumption of processed foods, as these can be harmful to health. |  |
|  |  |  |  |  |
|  |  |  |  |  |
| **Lifestyle** | | | |  |
| Mental and psychological strength | e1 | Posts that address mental and psychological strength. | A list of measures to promote motivation to eat a carnivorous diet. |  |
|  |  |  |  |  |
| Exercise and movement | e2 | Posts that discuss sporting activities and exercise without visually depicting them. | The influencer reports that they went to the gym in the morning. |  |
|  |  |  |  |  |
| Health-related recommendations | e3 | Lifestyle recommendations that are intended to promote health and do not explicitly refer to the carnivorous diet and exercise. | The influencer advises against using toothpaste, as it is not necessary on a carnivorous diet. |  |
|  |  |  |  |  |
|  |  |  |  |  |
| **Advertising** | | | |  |
| Media formats | f1 | Recommendation for a media format, even if it is not labeled as advertising. In this category, a distinction can be made between different formats. | The influencer recommends a podcast on the topic of carnivorous nutrition. |  |
|  |  |  |  |  |
|  |  |  |  |  |
| Dietary supplements | f2 | A positive reference to a company that manufactures dietary supplements, even if it is not explicitly labeled as advertising. | The influencer shows which company's collagen supplements they use and says that they think the products are great. |  |
|  |  |  |  |  |
|  |  |  |  |  |
| Food  (-producers) | f3 | Positive references to a food product, a specific brand, or a food producer, excluding dietary supplements, even if these are not explicitly labeled as advertising. | The influencer films themselves consuming a food product and identifies the brand. |  |
|  |  |  |  |  |
|  |  |  |  |  |
| Body care products | f4 | Products that can be used for personal care. A post is also classified in this category if it is not explicitly labeled as advertising. | An influencer describes that they use an additive-free sunscreen. They show the brand of the cream. |  |
|  |  |  |  |  |
|  |  |  |  |  |
| Coaching and conselling programs | f5 | Content that positively mentions a specific coaching program or nutritional advice. A post is also classified in this category if it is not explicitly marked as advertising. | The influencer recommends their coaching on the topic of carnivorous nutrition. |  |
|  |  |  |  |  |
|  |  |  |  |  |
| Other | f6 | Entities that place the brand of another consumer product in a positive context. A post also falls into this category if it is not explicitly marked as advertising. | The influencer shows that they are using a new vacuum cleaner from a specific brand and emphasizes how quiet it is. |  |
|  |  |  |  |  |
|  |  |  |  |  |
| **Other** | | | |  |
| Other | g | All posts that are irrelevant to the data collection in terms of content. In other words, posts that are not related to the other main categories. | A picture of a sunrise without a relevant description. |  |
|  |  |  |  |  |

***Table S2*** *Overview of the main categories with coding numbers for the subcategories and the corresponding detailed categories.*

| **Main category** | **Subcategory** | **Detailed categories** |  |
| --- | --- | --- | --- |
|  |  |  |  |
| **Body, movement, and exercise** | a1 | Upper body, buttocks, abdomen, entire body |  |
|  | a2 | Chest, abdomen, buttocks, arms, shoulders, legs |  |
|  | a3 | Endurance training, strength training, exercise |  |
|  | a4 | Aging, hair, skin, nails |  |
| **Food** | b1 | Breakfast, lunch, snacks, dinner, undefined |  |
|  | b2 | Videographic, photographic |  |
|  | b3 | Recipe, frying pan, oven, pot, hot air fryer, hot coals, waffle iron, grill |  |
|  | b4 | Red meat, white meat, meat, bones, processed meat, animal fats, animal broth, offal, fish, seafood, shellfish, eggs, dietary supplements, dairy products |  |
|  |  |  |  |
| **Nutrition** | c1 | Weight loss/obesity, vitamin deficiency, eating disorders, transition to a carnivorous diet, wounds, polycystic ovary syndrome, migraines, chronic diseases, autoimmune diseases, genetic diseases, diabetes mellitus 1/2, arthritis/joint pain, infertility, other. Digestive problems, chronic inflammatory bowel disease, coronary heart disease, menstruation, inflammation, mental disorders, brain fog, other, doubts about scientific criticism, non-alcoholic fatty liver disease |  |
|  |  |  |  |
|  |  |  |  |
|  |  |  |  |
|  |  |  |  |
|  | c2 | General health, eyes, kidneys, heart, brain, liver, bones and teeth, immune system, increased energy, sexual function, endocrine system, sleep, digestion, mental health, ketosis, protection against oxidative stress, cravings, satiety, blood glucose |  |
|  |  |  |  |
|  | c3 | Post-workout, pre-workout, endurance, testosterone, protein quality and quantity, recovery, muscle growth |  |
|  |  |  |  |
|  | c4 | No need, Individual need, Other, Protein powder, Vitamin D, Magnesium, Omega-3 fatty acids, Electrolytes, Creatine |  |
|  |  |  |  |
| **Politics and society** | d1 | Neglect of climate change, food waste, animal ethics, animal production/agricultural systems |  |
|  |  |  |  |
|  | d2 | Rated as inexpensive, rated as expensive, no rating |  |
|  | d3 | Vegetarianism (negative), vegetarianism (neutral), ketovore, low fat, ketogenic, low carb, animal-based diet, paleo diet, |  |
|  |  |  |  |
|  | d4 | Carnivore content, non-carnivore content |  |
|  | d5 | Science, food industry, pharmaceutical industry, politics, media, (highly) processed foods, plant-based foods, restrictive diets, carbohydrates, fiber, chemicals/toxins |  |
|  |  |  |  |
| **Lifestyle** | e1 | Self-love, optimism, motivation, stress, not overloaded with negativity, minimalism |  |
|  | e2 | Endurance training, strength training, exercise, training in general |  |
|  | e3 | Cultivating interpersonal relationships, sunbathing, listening to your body, sleep rhythm, other nutritional recommendations, frequency healing, cold showers, meditation, tracking macronutrients, alcohol consumption, fasting, meal prep, infrared light, not weighing yourself too often. |  |
|  |  |  |  |
| **Advertising** | f1 | YouTube, book, Patreon, website, documentary, podcast |  |
|  | f2 | Electrolytes, miscellaneous, enzymes, collagen, brand advertising, vitamins/minerals, protein powder |  |
|  | f3 | Salt, spices, meat processing plants, milk processing plants, discount stores, Nova 1, Nova 2, Nova 3, Nova 4, fast food chain/restaurant |  |
|  |  |  |  |
|  | f4 | Skin and hair care products, deodorant, red light therapy |  |
